# B‐Lymphoblastic Leukemia Presenting as Bilateral Ovarian Masses in a 20‐Year‐Old Woman

**DOI:** 10.1002/ccr3.73736

**Published:** 2026-10-17

**Authors:** Jennifer Cai, David Sin, Marian Varda, Lauren Lewis, James Yeh

**Affiliations:** ^1^ University of California Irvine Irvine California USA; ^2^ Department of Radiology Harbor‐UCLA Medical Center Torrance California USA; ^3^ Division of Hematology and Medical Oncology, Department of Medicine Harbor‐UCLA Medical Center Torrance California USA; ^4^ David Geffen School of Medicine at UCLA Los Angeles California USA

**Keywords:** B‐ALL, B‐LBL, B‐lymphoblastic leukemia, B‐lymphoblastic lymphoma, leukemia, lymphoma, ovarian cancer

## Abstract

Hematologic malignancies involving the ovaries are rare and may mimic ovarian carcinoma. We report a 20‐year‐old woman who presented with generalized body pain, bilateral ovarian masses, and abdominal adenopathy, initially suspected to have ovarian carcinoma. Despite normal blood counts, a retroperitoneal lymph node biopsy and subsequent bone marrow biopsy confirmed B‐lymphoblastic leukemia. Molecular analysis identified multiple pathogenic alterations, including *CREBBP*, *KMT2D*, and *KRAS* mutations, *CDKN2A* copy number loss, and *FLT3* overexpression. The patient developed tumor lysis syndrome and was transferred to a tertiary center, after which she was lost to follow‐up. While long‐term treatment response could not be evaluated, this case underscores the clinical necessity of including B‐lymphoblastic leukemia/lymphoma in the differential diagnosis of bilateral ovarian masses in young women, even with normal peripheral blood counts. Early tissue diagnosis through minimally invasive biopsy is essential to avoid unnecessary surgery and to initiate appropriate systemic therapy.

## Key Clinical Message

While lymphoid neoplasms rarely involve the ovaries, B‐lymphoblastic leukemia/lymphoma (B‐ALL/LBL) should be considered in adolescents and young women with ovarian masses. A normal CBC does not exclude B‐ALL; in addition to tissue biopsy, a peripheral blood smear review is a low‐cost, high‐yield diagnostic step that helps avoid unnecessary oophorectomy.

## Graphical abstract

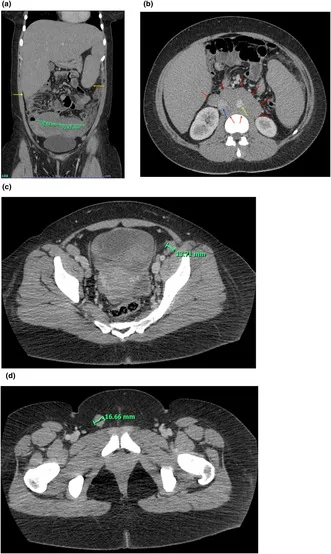

## 1 Introduction

Ovarian masses are common among women of all ages. While many remain asymptomatic and unidentified, others can cause symptoms such as abdominal distention, pelvic pain or pressure, changes in menstrual cycles, or gastrointestinal or urinary issues. These masses can vary in size, shape, and characteristics. Although the majority (80%–85%) of ovarian masses are benign, malignant ovarian tumors can be life‐threatening if not detected early [1]. Despite accounting for less than one‐third of cancers in the female reproductive system, ovarian cancer is responsible for two‐thirds of gynecologic cancer‐related deaths [2]. Early detection and thorough evaluation are vital to differentiate between benign and malignant ovarian masses, ensuring the timely implementation of appropriate treatment. Key factors for predicting whether an ovarian mass is benign or malignant include the patient's age, family history, menopausal status, symptoms, physical examination findings, imaging results, and the blood level of the cancer biomarker cancer antigen (CA) 125 [1].

The majority of ovarian malignancies originate from epithelial cells and predominantly affect postmenopausal women. Hematologic malignancies presenting with ovarian involvement are rare and may closely mimic primary ovarian neoplasms both clinically and radiographically [3, 4]. The most common histological subtypes of lymphoma involving the ovaries include diffuse large B‐cell lymphoma, follicular lymphoma, Burkitt lymphoma, and extranodal marginal zone lymphoma of mucosa‐associated lymphoid tissue [5].

B‐lymphoblastic leukemia/lymphoma (B‐ALL/LBL) is a precursor B‐cell malignancy most common in pediatric and young adult populations. Though it frequently involves the bone marrow, peripheral blood, and various extramedullary sites [e.g., central nervous system (CNS), testes, liver, spleen, and skin], the ovaries are rarely affected. The nomenclature is determined by the primary site of involvement: B‐ALL is characterized by predominant bone marrow and peripheral blood disease, whereas B‐LBL describes cases where the malignancy is localized to lymph nodes or other extramedullary organs.

In this report, we describe the case of a 20‐year‐old female with no significant medical or family history, who presented with generalized body pain and elevated CA 125. Radiographic imaging demonstrated bilateral ovarian masses, highly suspicious for ovarian carcinoma. However, subsequent histologic examination unexpectedly revealed B‐ALL. Because the clinical and radiographic features were suggestive of ovarian carcinoma, a hematologic malignancy was not initially suspected. Consequently, the patient was managed on a standard outpatient solid‐tumor diagnostic timeline, which created a 19‐day diagnostic interval. This delay underscores the primary teaching point of this report: B‐ALL/LBL can perfectly mimic advanced gynecologic carcinoma, successfully misdirecting clinicians until tissue biopsy reveals the true underlying pathology. To date, fewer than 10 cases of B‐ALL/LBL presenting as ovarian masses at initial diagnosis have been reported in the English literature [6, 7, 8, 9, 10, 11, 12, 13, 14]. This case highlights a rare but important diagnostic consideration in the evaluation of ovarian masses in young patients, where misdiagnosis can lead to unnecessary, non‐therapeutic surgical intervention.

Written informed consent was obtained from the patient for publication of this case report and accompanying radiographic and pathologic images.

## 2 Case History and Examination

A 20‐year‐old nulligravid female with no significant past medical history presented to the emergency department of a large, urban, safety‐net tertiary academic medical center on Day 0 with generalized body pain. She had visited the emergency department of a community hospital the previous day, where a computed tomography (CT) scan revealed large bilateral adnexal/ovarian masses, bulky intra‐abdominal adenopathy, and peritoneal thickening, highly suspicious for ovarian carcinoma with metastases. At the medical center, she denied fever, chills, nausea, vomiting, diarrhea, constipation, dysuria, hematuria, vaginal discharge/bleeding, weight changes, or night sweats. Her family history was negative for gynecologic malignancies.

The physical examination revealed bilateral scleral icterus and hepatosplenomegaly, with no other significant findings.

### 2.1 Diagnostic Workup

At the presentation (Day 0), a complete blood count (CBC) showed no significant abnormalities [white blood cell (WBC) 7.8 K/cumm; hemoglobin 13.3 g/dL; platelet 310 K/cumm]. She was found to have mildly elevated CA 125 (56.5 U/mL, reference range: 0.0–35.0 U/mL), markedly elevated lactate dehydrogenase (LDH, 869 U/L, reference range: 98–192 U/L), and significantly elevated liver enzymes and bilirubin [aspartate aminotransferase (AST, 157 U/L, reference range: 15–41 U/L), alanine aminotransferase (ALT, 289 U/L, reference range: 7–35 U/L), alkaline phosphatase (171 U/L, reference range: 38–126 U/L), total and direct bilirubin (1.6 and 0.8 mg/dL, reference range: 0.36–1.2 and 0.1–0.5 mg/dL, respectively)], without elevation of carbohydrate antigen (CA) 19‐9, carcinoembryonic antigen (CEA), alpha‐fetoprotein (AFP), or human chorionic gonadotropin (hCG). Transvaginal ultrasound identified bilateral hypoechoic solid, smooth masses with very strong flow, which correspond to Ovarian‐Adnexal Reporting and Data System (O‐RADS) 5 adnexal masses (right 8.5 cm; left 9.9 cm) with internal vascularity (Figure 1). She was admitted on Day 19 for further diagnostic work and treatment. At admission (Day 19), CBC was still within normal reference ranges (WBC 7.7 K/cumm; hemoglobin 13.0 g/dL; platelet 164 K/cumm) but revealed a decreased platelet count relative to her initial presentation. CT was repeated on Day 19, demonstrating large soft tissue masses in the bilateral adnexa measuring approximately 7.5 and 7.4 cm in diameter, with diffuse bulky retroperitoneal lymphadenopathy in the periaortic and pericaval regions and scattered prominent lymph nodes in the lower abdomen/pelvis and inguinal region (Figure 2). Significant hepatosplenomegaly and inflammatory stranding of the omentum were also noted. Magnetic resonance imaging (MRI) of the brain did not identify any evidence of intracranial metastases.

**FIGURE 1:**
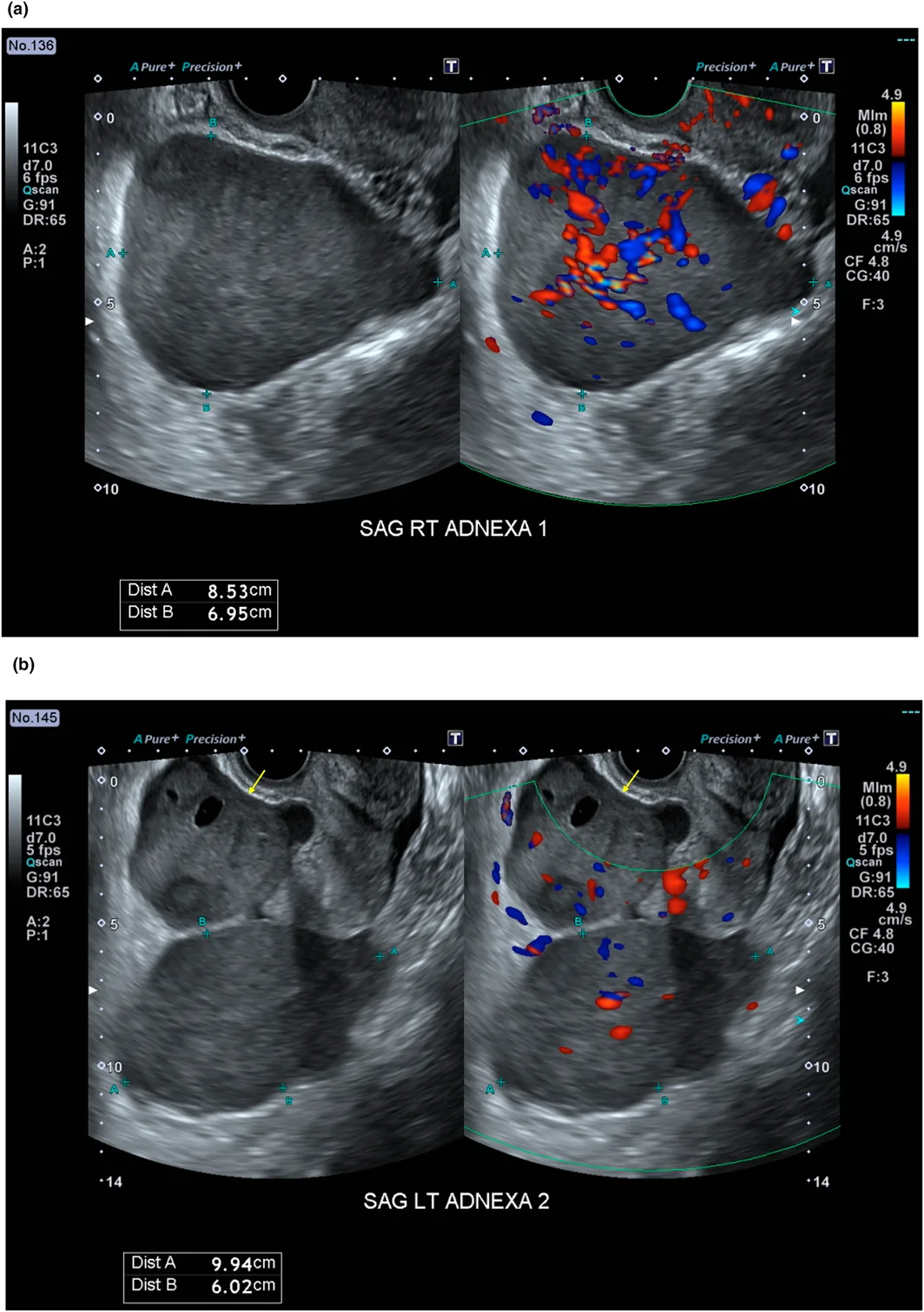
(a) Sagittal grayscale (left image) and color Doppler (right image) transvaginal ultrasound of the right adnexa shows a hypoechoic solid, smooth mass (calipers) with very strong flow, which corresponds to O‐RADS 5. The mass measures 8.5 cm in maximal dimension. (b) Sagittal grayscale (left image) and color Doppler (right image) transvaginal ultrasound of the left adnexa shows a similar hypoechoic solid, smooth mass (calipers) measuring 9.9 cm in maximal dimension. The mass demonstrates very strong flow (not shown) and is scored O‐RADS 5. In addition, the right adnexal mass (yellow arrow) is also visible superficially.

**FIGURE 2:**
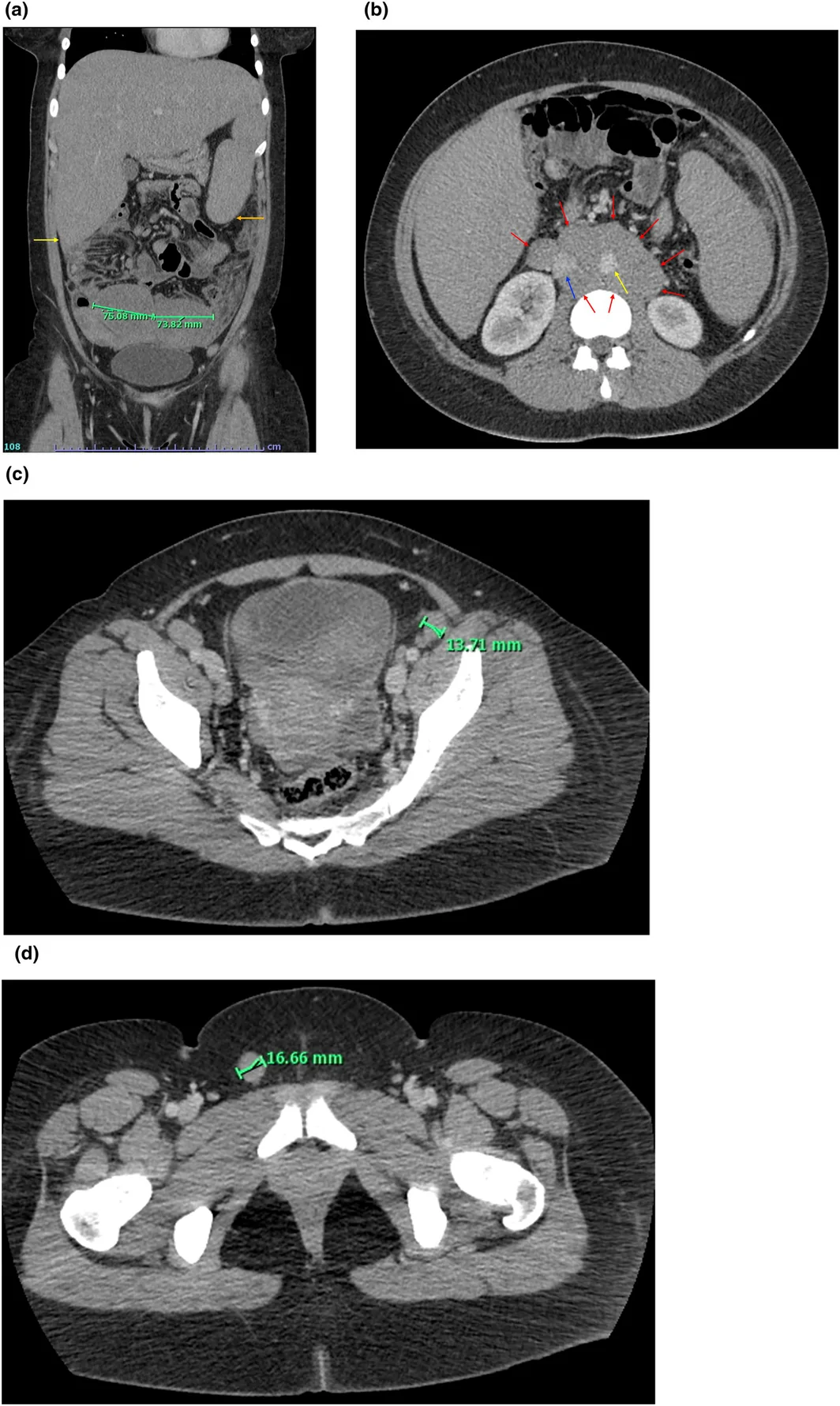
(a) Coronal reformat computed tomography (CT) abdomen/pelvis with IV contrast shows bilateral adnexal masses (annotated on image). A subtle hypoattenuating fat plane (red arrow) separates the masses, which are soft tissue attenuation and hypoenhancing relative to an enlarged liver (yellow arrow). There is also partially visualized splenomegaly (orange arrow). (b) Axial CT abdomen/pelvis with IV contrast at the level of the kidneys shows confluent retroperitoneal lymphadenopathy (red arrows) encasing the contrast‐enhanced aorta (yellow arrow) and IVC (blue arrow). (c) Axial CT abdomen/pelvis with IV contrast at the level of the bladder dome shows a mildly enlarged left external iliac lymph node (annotated on image) measuring 14 mm in short axis. (d) Axial CT abdomen/pelvis with IV contrast slightly lower at the level of the pubic symphysis shows a mildly enlarged right inguinal lymph node (annotated on image) measuring 17 mm in short axis.

A CT‐guided core biopsy of the retroperitoneal lymph node (Figure 3) was performed on Day 22. This approach was preferred to a direct ovarian biopsy to minimize the risk of ovarian capsule rupture and subsequent intraperitoneal tumor cell seeding, while simultaneously achieving both tissue diagnosis and advanced nodal staging. Histopathological analysis revealed that the lymph node architecture was completely effaced by a diffuse infiltrate of lymphoid blasts. These blasts were intermediate in size and exhibited immature nuclear chromatin. Immunohistochemical stains demonstrated that the blasts were positive for PAX‐5, CD10, BCL2, CD34 (subset), and TdT, with a high proliferation rate as indicated by Ki67 (90%). The blasts were negative for CD3, CD20, and BCL6. The findings were consistent with a diagnosis of B‐ALL/LBL. Given the frequent involvement of peripheral blood in B‐ALL/LBL, the peripheral blood smear at admission was examined, showing 2% blasts. Additionally, a bone marrow biopsy performed on Day 26 revealed markedly hypercellular bone marrow (> 95%) with 60% B‐lymphoblasts, confirming the diagnosis of B‐ALL (Figure 4). At the time of the bone marrow biopsy, the peripheral blood showed pancytopenia (WBC 1.8 K/cumm; hemoglobin 9.6 g/dL; platelet 73 K/cumm) with 12% circulating blasts. Next‐generation DNA/RNA sequencing (Tempus Lab, Chicago, IL) performed on the bone marrow biopsy identified several genetic alterations, including a loss‐of‐function (LOF) mutation in *CREBBP* (p.R483*), a gain‐of‐function (GOF) mutation in *KRAS* (p.G12V), a frameshift (LOF) mutation in *KMT2D* (p.R661fs), *CDKN2A* copy number loss, and *FLT3* overexpression; no gene rearrangements nor altered splicing events were identified.

**FIGURE 3:**
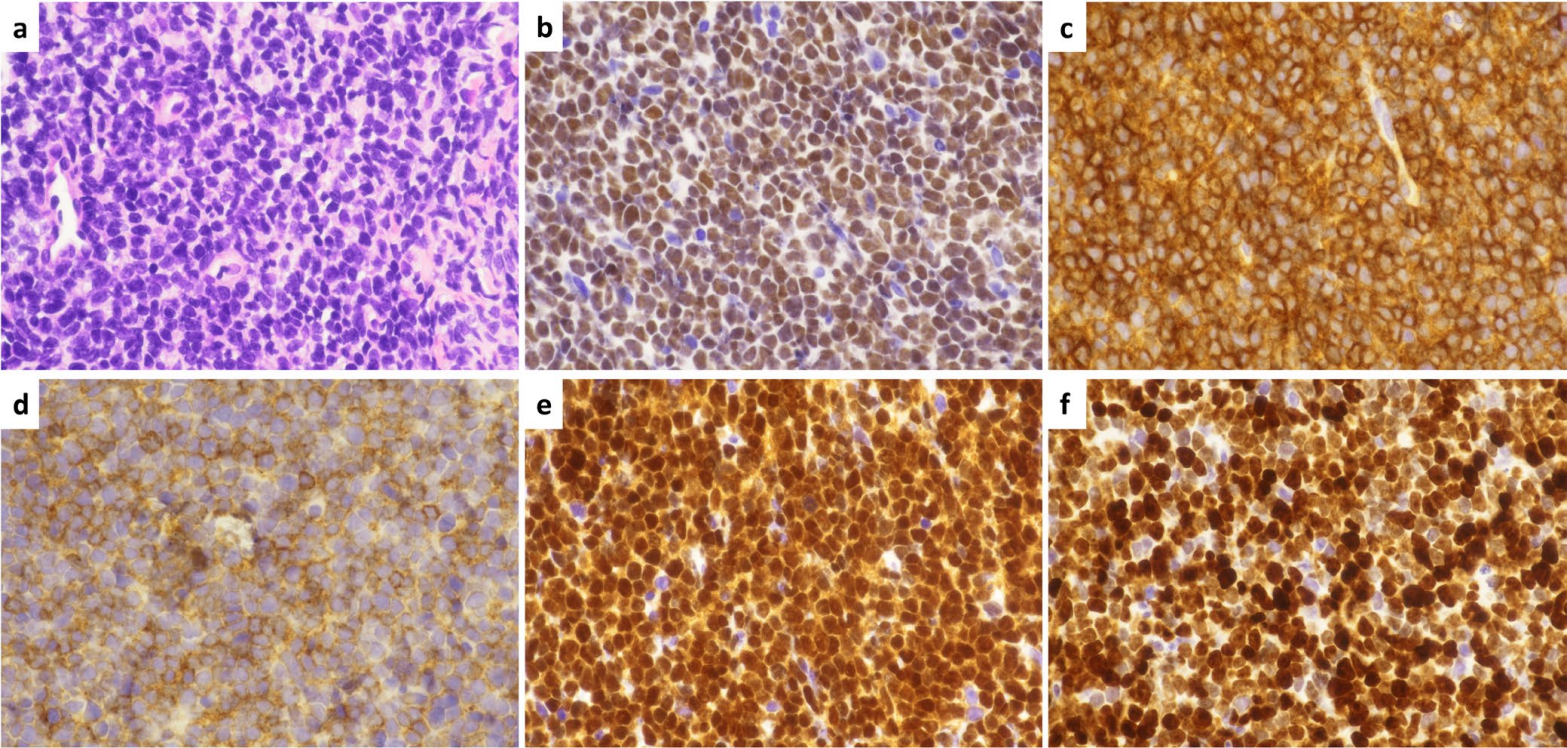
Computed tomography‐guided core biopsy of the retroperitoneal lymph node. (a) The lymph node architecture is effaced by a diffuse infiltrate of intermediate‐sized lymphoid blasts. (b–f) Immunohistochemical staining reveals that the blasts are positive for PAX‐5 (b), CD10 (c), BCL2 (not shown), CD34 (subset, d), and TdT (e), with a high proliferation rate as indicated by Ki67 (f). The blasts are negative for CD3, CD20, and BCL6 (not shown). The findings are consistent with B‐lymphoblastic leukemia/lymphoma (B‐ALL/LBL). (Original magnification, ×400].

**FIGURE 4:**
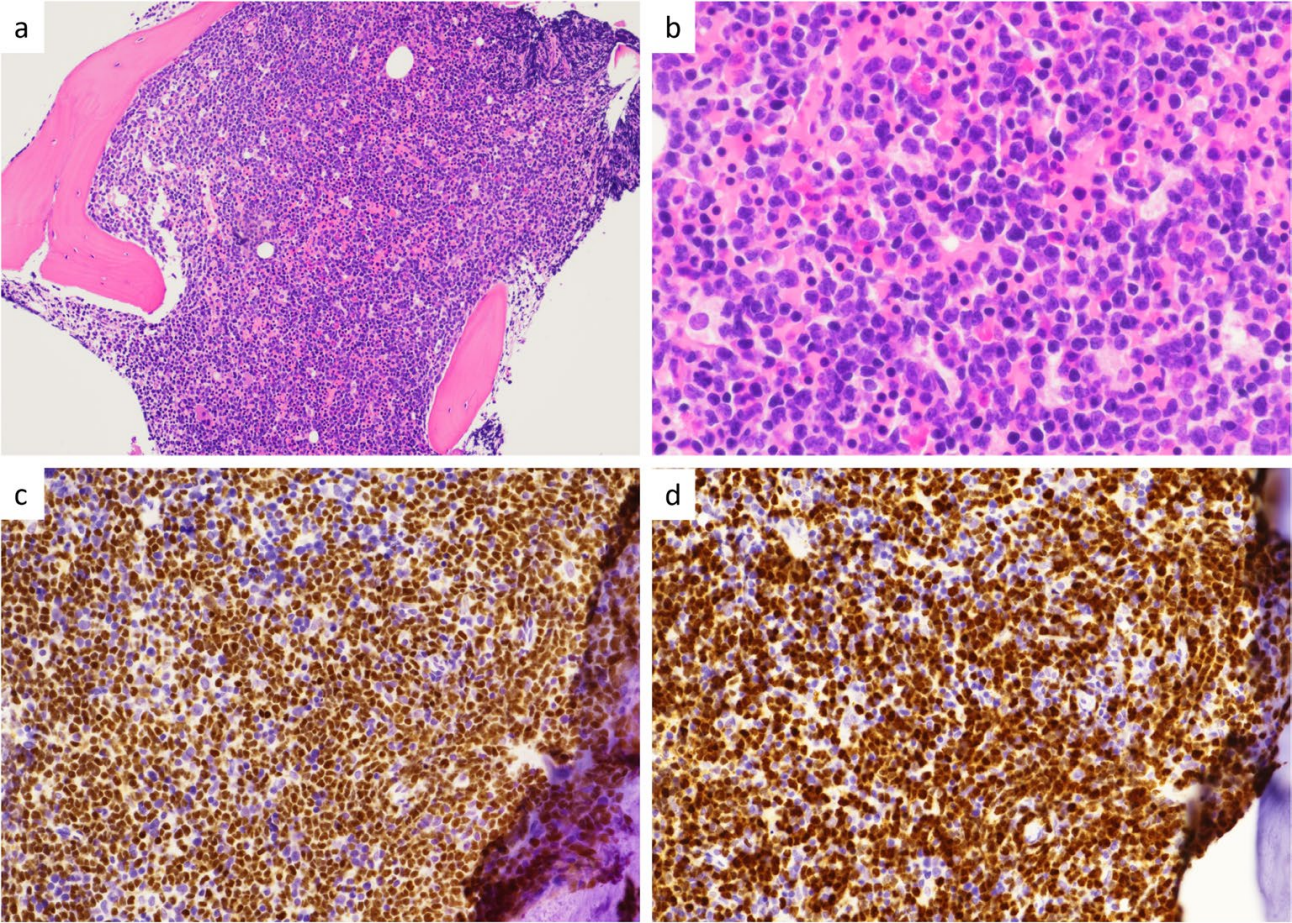
Bone marrow biopsy. (a, b) The bone marrow is markedly hypercellular (> 95%) with many blasts. (c, d) The blasts exhibit a similar immunohistochemical staining pattern as the lymph node biopsy. They are positive for PAX‐5 (c) and TdT (d) and represent approximately 60% of bone marrow cellularity [original magnification, ×100 (a), ×400 (b), × 200 (c,d)].

## 3 Outcome

While awaiting chemotherapy, the patient developed tumor lysis syndrome on Day 25. Her condition improved following treatment with bicarbonate infusion and rasburicase. On Day 27, she was transferred to a tertiary Children's Hospital for further management of her tumor lysis syndrome, as well as for evaluation for a bone marrow transplant and potential enrollment in a clinical trial, after which she was lost to long‐term oncologic follow‐up due to inter‐institutional data‐sharing constraints.

## 4 Discussion

Although ovarian involvement by lymphoma or leukemia is rare, distinguishing these entities from other ovarian malignancies is crucial, as their management and prognosis differ significantly. While most lymphomas affect both ovaries [15], they account for only a small fraction of bilateral ovarian masses, with the majority of such cases being attributed to serous carcinoma, mature teratoma, or metastasis [16]. The diagnosis of lymphomas and leukemias involving the ovaries relies primarily on histological evaluation. This case emphasizes the need to include lymphoma/leukemia in the differential diagnosis of bilateral ovarian masses in adolescents and young women, as early diagnosis can significantly impact management decisions, including preventing unnecessary oophorectomy. Unlike most lymphomas, which typically affect women in their 40s to 60s, Burkitt lymphoma and B‐ALL/LBL often present in children and young adults. Clinically, ovarian involvement by B‐ALL/LBL may resemble other lymphomas presenting as ovarian tumors. However, B‐ALL/LBL more commonly manifests as a systemic disease with bone marrow and peripheral blood involvement, whereas other lymphomas often initially present as localized ovarian masses without marrow infiltration. Searching for blasts in the peripheral blood can aid in diagnosis. In cases where the peripheral blood is not involved, conducting a mass or bone marrow biopsy can provide valuable diagnostic insights. While needle biopsies are typically avoided in ovarian cancer due to the risk of spreading cancer cells, they may be considered in young patients when imaging and clinical findings do not strongly suggest primary ovarian epithelial malignancies. In cases with diffuse lymphadenopathy, like the one presented here, a preoperative lymph node biopsy may be beneficial.

The present case highlights an unusual presentation of B‐ALL as bilateral large ovarian masses. Ovarian involvement in B‐ALL/LBL is uncommon, and when it does occur, it usually represents an ovarian relapse of previously diagnosed leukemia [17, 18, 19]. As noted previously, 10 cases of B‐ALL/LBL presenting as ovarian masses at initial diagnosis have been reported in the literature [6, 7, 8, 9, 10, 11, 12, 13, 14]; as outlined in Table 1, our case represents a rare presentation of B‐ALL with bilateral masses, contrasting with the predominantly unilateral B‐ALL presentations or the TCF3::PBX1‐association found in the literature. Except for features such as young age and highly elevated serum LDH, the clinical, laboratory, and radiographic findings of these reported cases often resembled those of other ovarian malignancies, such as ovarian carcinoma. In our patient's case, the combination of young age, diffuse lymphadenopathy, and markedly elevated LDH could have raised suspicion for lymphoma, particularly B‐ALL/LBL or Burkitt lymphoma. However, the initial absence of peripheral blood involvement and the lack of bone marrow failure (evidenced by anemia, neutropenia, and/or thrombocytopenia) posed a significant diagnostic challenge.

**TABLE 1:** Summary of B‐lymphoblastic leukemia/lymphoma cases presenting as ovarian masses at initial diagnosis.

References	Age (Y)	Presentation	Ovarian involvement	Lymphadenopathy	Diagnostic methods	Final diagnosis	Key lab/Genetic findings	Treatment	Clinical outcome
Maitra et al. [6]	7	Abdominal mass	N/A	N/A	Ovarian tissue; unknown method	B‐LBL	N/A	Chemotherapy	CR (3‐month follow‐up)
Iyengar et al. [7]	46	Increased abdominal girth	Bilateral	N/A	TAH‐BSO + omental sampling	B‐LBL	N/A	Surgery + Chemotherapy	Alive (relapsed in bone 6 months later, 8 months follow‐up)
Sakurai et al. [8]	19	Urinary tract infection	Bilateral	Yes	Right salpingo‐oophorectomy	B‐LBL	N/A	Surgery + Chemotherapy	CR (1‐year follow‐up)
Zhao et al. [9]	N/A	Pelvic mass	N/A	N/A	Unilateral oophorectomy or TAH‐BSO	B‐LBL	N/A	Surgery + Chemotherapy	Survived 14.1 months
Yadav et al. [10]	28	Incidental finding	Bilateral	No	TAH‐BSO + omentectomy	B‐LBL	Elevated LDH, normal CA125	No	Leukemic transformation after 7 months, expired before chemotherapy
Narayanan and Soman [11]	15	Abdominal pain	Bilateral	Yes	Ovarian Tru‐Cut biopsy	B‐LBL	Elevated LDH	Chemotherapy	CR (10‐year follow‐up)
Joel et al. [12]	18	Abdominal pain	Unilateral (left)	No	Ovariectomy	B‐LBL	N/A	Surgery + Chemotherapy	CR (2‐year follow‐up)
Kubota‐Tanaka et al. [13]	15	Coxalgia	Bilateral	N/A	N/A	B‐LBL	Elevated LDH, *TCF3::PBX1* Fusion Gene	Chemotherapy	Resistant to treatment/Early relapse
Vinti et al. (Case 1) [14]	12	Abdominal pain	Unilateral (left)	N/A	Bone marrow biopsy	B‐ALL	*TCF3::PBX1* Fusion Gene	Chemotherapy + Surgery	Resistant to treatment/Early relapse
Vinti et al. (Case 2) [14]	5	Abdominal pain	Unilateral (right)	N/A	Ovarian biopsy + bone marrow biopsy	B‐ALL	*TCF3::PBX1* Fusion Gene	Chemotherapy	Resistant to treatment/Early relapse
Current case	20	Generalized body pain	Bilateral	Yes	Lymph node biopsy + bone marrow biopsy	B‐ALL	Elevated LDH, Elevated CA 125	N/A	Lost to follow‐up

Abbreviations: B‐ALL, B‐lymphoblastic leukemia; B‐LBL, B‐lymphoblastic lymphoma; CR, complete remission; N/A, information not available; TAH‐BSO + omental sampling, total abdominal hysterectomy, bilateral salpingo‐oophorectomy.

A normal CBC despite significant bone marrow infiltration in B‐ALL is a well‐recognized phenomenon [20]. It may occur in early‐stage disease when compensatory hematopoiesis remains preserved and when leukemic blasts are biologically retained within the marrow microenvironment. Retention of lymphoblasts in the bone marrow may be mediated by the high‐affinity binding of integrins such as VLA‐4 to stromal ligands like VCAM‐1 [21], alongside an intact marrow‐blood barrier that prevents the egress of blasts into the peripheral circulation. Furthermore, the chemokine signaling through the CXCL12/CXCR4 axis may play a role in trapping CXCR4‐positive blasts within marrow stem cell niches [22] and temporarily allowing non‐infiltrated areas to maintain normal hematopoiesis. These mechanisms may have delayed the appearance of circulating blasts and cytopenias in our patient. At the presentation, this patient's CBC showed no abnormalities. On the day of admission (Day 19), her CBC was still within normal limits but revealed a decreased platelet count. Additionally, 2% blasts were identified in her peripheral blood smear at admission. It is not clear if circulating blasts were present initially, as a peripheral blood smear was not made at that time. Seven days after her admission, on Day 26, the CBC showed pancytopenia with 12% blasts in the peripheral blood smear. These findings indicate rapid disease progression and suggest that monitoring CBCs and searching for blasts in peripheral blood smears may facilitate diagnosis in the appropriate clinical context. To address this diagnostic challenge, a peripheral blood smear should be obtained early in young patients presenting with bilateral ovarian masses and diffuse lymphadenopathy, even when the initial automated CBC is completely normal.

A limitation of this case report is the lack of direct histological confirmation from the ovaries, as tissue diagnosis was established solely via lymph node biopsy. Therefore, the theoretical possibility of a coincidental occurrence of B‐ALL in the lymph nodes alongside a separate primary ovarian neoplasm cannot be completely excluded. However, synchronous primary malignancies involving B‐ALL and an unrelated solid tumor are exceedingly rare. Moreover, several clinical and radiographic factors in our patient strongly argue against synchronous primary malignancies. Specifically, the bilateral ovarian masses and the involved lymph nodes demonstrated identical radiographic features on CT, strongly pointing to a single, systemic disease process. Additionally, her serum tumor marker profile provides robust evidence against a coincident epithelial ovarian neoplasm. Her CA‐125 was only mildly elevated, a nonspecific finding that can be observed in many conditions, including hematologic malignancies [23]. Her completely normal CA19‐9 and CEA levels ruled out advanced gastrointestinal cancers or ovarian mucinous neoplasms. Additionally, normal AFP and hCG levels made germ cell tumors, which are common in the age group of our patient, unlikely.

In our case, next‐generation DNA/RNA sequencing of the bone marrow biopsy revealed several genetic alterations, including LOF mutations in *CREBBP* (p.R483*) and *KMT2D* (p.R661fs), a GOF mutation in *KRAS* (p.G12V), *CDKN2A* copy number loss, and *FLT3* overexpression. However, no gene rearrangement, including the previously reported *TCF3::PBX1* fusion gene [18], or altered splicing events were identified in our patient. LOF mutations in *CREBBP* have been associated with high‐risk disease and chemoresistance in B‐ALL [24]. Ras pathway mutations are prevalent in relapsed childhood ALL, and *KRAS* GOF mutations correlate with poorer survival [25]. LOF mutations of *KMT2D*, like the frameshift detected in our patient, contribute to the silencing of critical tumor suppressor genes that control B cell activating pathways [26], predisposing them toward malignancy. However, the precise role of *KMT2D* in B‐ALL remains incompletely defined [27]. In B‐ALL, clones harboring *CDKN2A/B* deletions at initial diagnosis consistently persist through disease recurrence, and the prevalence of these deletions significantly increases in relapsed cases [28]. These findings, corroborated by Ampatzidou et al. [29], suggest that *CDKN2A/B* deletions may serve as a significant prognostic indicator for relapse and inferior overall survival in B‐ALL. Finally, *FLT3* overexpression was documented across all B‐ALL subtypes, with the highest expression levels in *ZNF384*‐rearranged, *KMT2A*‐rearranged, and hyperdiploid cases [30]. However, Gutierrez‐Camino et al. observed no association between *FLT3* expression levels and risk parameters (e.g., WBC, CNS involvement, minimal residual disease status, and relapse) or survival outcomes [30]. The genetic alterations identified in our patient likely accounted for the rapid clinical progression observed. While somatic mutations such as CREBBP and KRAS do not independently dictate a referral for allogeneic bone marrow transplantation under current guidelines, the highly aggressive clinical presentation of this case—marked by bulky extramedullary ovarian masses and diffuse lymphadenopathy—would clinically prompt an early transplant evaluation.

In conclusion, this case highlights the diagnostic complexity of ovarian masses in young patients and emphasizes the importance of considering hematologic malignancies even in the absence of peripheral blood abnormalities. The loss to follow‐up limits our ability to comment on long‐term outcomes, but the diagnostic and therapeutic principles illustrated remain instructive. Prompt tissue diagnosis, integration of clinical and laboratory findings, and molecular characterization are essential to ensure appropriate treatment and avoid unnecessary surgical intervention.

## Author Contributions

**Jennifer Cai:** conceptualization, investigation, methodology, project administration, resources, visualization, writing – original draft, writing – review and editing. **David Sin:** methodology, resources, visualization, writing – original draft, writing – review and editing. **Marian Varda:** investigation, project administration, resources, writing – review and editing. **Lauren Lewis:** project administration, resources, writing – review and editing. **James Yeh:** investigation, project administration, resources, writing – review and editing.

## Funding

The authors have nothing to report.

## Ethics Statement

The authors have nothing to report.

## Consent

Written informed consent was obtained from the patient to publish this report in accordance with the journal's patient consent policy.

## Conflicts of Interest

The authors declare no conflicts of interest.

## Data Availability

No datasets were generated or analyzed during the current study.
